# Role of inflammasomes in the pathogenesis of periodontal disease and therapeutics

**DOI:** 10.1111/prd.12269

**Published:** 2019-12-18

**Authors:** Julie T. Marchesan, Mustafa Saadat Girnary, Kevin Moss, Eugenia Timofeev Monaghan, Grant Joseph Egnatz, Yizu Jiao, Shaoping Zhang, Jim Beck, Karen V. Swanson

**Affiliations:** ^1^ Department of Periodontology Adams School of Dentistry University of North Carolina Chapel Hill North Carolina, USA; ^2^ Department of Oral and Craniofacial Health Sciences Adams School of Dentistry University of North Carolina Chapel Hill North Carolina, USA; ^3^ Periodontics Department College of Dentistry University of Iowa Iowa City Iowa, USA; ^4^ Department of Dental Ecology Adams School of Dentistry University of North Carolina Chapel Hill North Carolina, USA; ^5^ Department of Medicine, Infectious Disease University of North Carolina at Chapel Hill Chapel Hill North Carolina, USA

## Abstract

Inflammasomes are a group of multimolecular intracellular complexes assembled around several innate immune proteins. Recognition of a diverse range of microbial, stress and damage signals by inflammasomes results in direct activation of caspase‐1, which subsequently induces the only known form of secretion of active interleukin‐1β and interleukin‐18. Although the importance of interleukin‐1β in the periodontium is not questioned, the impact of inflammasomes in periodontal disease and its potential for therapeutics in periodontology is still in its very early stages. Increasing evidence in preclinical models and human data strongly implicate the involvement of inflammasomes in a number of inflammatory, autoinflammatory and autoimmune disorders. Here we review: (a) the currently known inflammasome functions, (b) clinical/preclinical data supporting inflammasome involvement in the context of periodontal and comorbid diseases and (c) potential therapies targeting inflammasomes. To clarify further the inflammasome involvement in periodontitis, we present analyses of data from a large clinical study (n = 5809) that measured the gingival crevicular fluid‐interleukin‐1β and grouped the participants based on current periodontal disease classifications. We review data on 4910 European‐Americans that correlate 16 polymorphisms in the interleukin‐1B region with high gingival crevicular fluid‐interleukin‐1β levels. We show that inflammasome components are increased in diseased periodontal tissues and that the caspase‐1 inhibitor, VX‐765, inhibits ~50% of alveolar bone loss in experimental periodontitis. The literature review further supports that although patients clinically present with the same phenotype, the disease that develops probably has different underlying biological pathways. The current data indicate that inflammasomes have a role in periodontal disease pathogenesis. Understanding the contribution of different inflammasomes to disease development and distinct patient susceptibility will probably translate into improved, personalized therapies.

## 1 INTRODUCTION

The innate immune response is the body's first line of defense against pathogens. The innate immune system recognizes pathogens, including bacteria and viruses, by engagement of the germline encoded pattern recognition receptors (PRR). There are five families of PRRs that are able to sense a vast array of microbial components, referred to as pathogen‐associated molecular patterns (PAMP) and damage‐associated molecular patterns (DAMP), that are host cell components produced during inflammation or environmentally derived, such as exposure to silica. Although PRRs are predominately expressed by innate immune cells, many of the PRRs are also found on other cells, including epithelial, endothelial and cells of the adaptive immune system. PRR engagement by its ligand induces downstream signaling cascades that induce multiple effects, including activation of innate immune cells and cytokine/chemokine production for the recruitment of immune cells to the site of infection or tissue damage.

**Figure 1:**
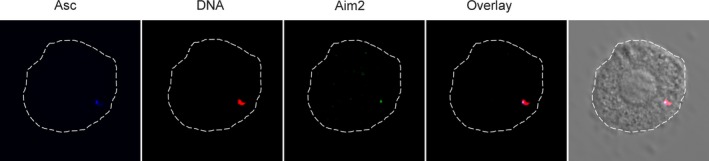
Visualization of inflammasome activation by recognition of cytosolic DNA. Murine dendritic cells were lipopolysaccharide primed and stimulated with rhodamine‐labeled poly‐dAdT DNA, resulting in Aim2 inflammasome activation. Confocal images show an overlay of pseudo‐colored ASC (blue), DNA (red) and Aim2 (green) in the cytosol of a cell. Methods described in Swanson et al^27^

A key function of the innate immune system is inflammasome activation. In response to PAMPs or DAMPS, some PRRs assemble inflammasomes (Figure 1) for the activation of cellular caspases that, in turn, induce the maturation of the proinflammatory cytokines interleukin‐1β and interleukin‐18 together with the induction of inflammation‐induced programmed cell death (pyroptotic). Although it had been known since the early 1990s that caspase‐1 was able to cleave pro‐interleukin‐1β and trigger cell death (later termed pyroptosis in contrast to apoptosis), it was not until a decade later, with a seminal paper by Martinon et al^1^ that the details of how caspase‐1 is activated were unraveled with the discovery of the inflammasome.

**Figure 2:**
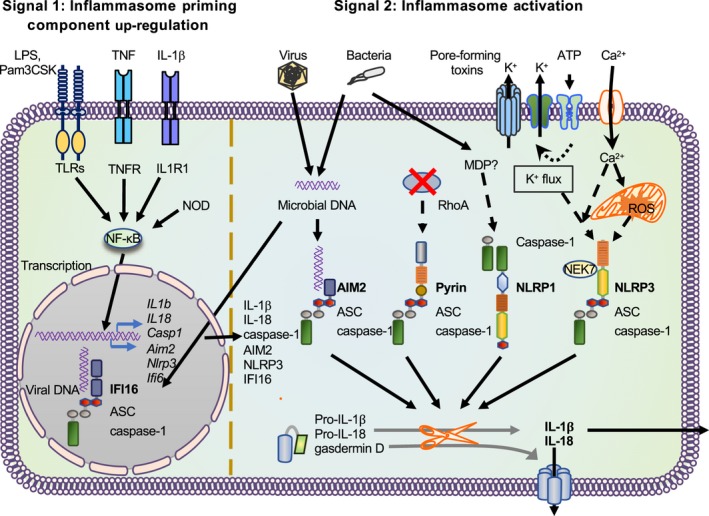
Inflammasome priming and activation. Inflammasomes must be primed (signal 1) before activation (signal 2). First, a nuclear factor‐κB‐activating stimulus, such as lipopolysaccharide or tumor necrosis factor‐β, induces elevated expression of inflammasome components (*IL1B*,*IL18*,*CAS1 AIM2*,*NLRP3*,*IFI16*), which leads to increased expression of these proteins. After priming, inflammasome activation requires a second, specific signal to activate each individual inflammasome and lead to the formation of unique inflammasome complexes. On receiving an activating signal, inflammasome sensors multimerize, and recruit ASC and pro‐caspase‐1, promoting the autoactivation of caspase‐1. Interleukin‐1β and interleukin‐18 are synthesized as proproteins that are processed by caspase‐1 into active forms and are secreted by cells. Although the mechanism(s) of secretion are not fully elucidated, some interleukin‐1β and interleukin‐18 secretion occurs via gasdermin D‐pores, as shown^172^

Inflammasomes are multimeric protein structures composed of a sensor molecule (the PRR), typically the adapter molecule apoptosis‐associated speck‐like protein containing a caspase‐recruitment domain (CARD), and the protease caspase‐1. There are multiple inflammasomes that can be formed, which are named for their sensor PRR that induces its activation. Inflammasome sensor molecules cross multiple PRR families, including a nucleotide‐binding domain, leucine‐rich repeat‐containing proteins (NLR, also known as NOD‐like receptors), absent in melanoma 2 (AIM2)‐like receptors (ALRs) and retinoic acid‐inducible gene I (Rig‐I)‐like receptors (RLR; Figure 2). Although inflammasomes are widely recognized to be activated in myeloid cells, including monocytes, macrophages, dendritic cells and neutrophils, they can also be activated in keratinocytes, gingival and dermal fibroblasts,^2^ and mucosal epithelial cells.

Inflammasome activation is a highly inflammatory process that is often initiated during pathogen infections (in communicable diseases) or during sterile inflammation by detection of proinflammatory debris (in noncommunicable diseases). Inflammasome activation induces the maturation of the proinflammatory cytokines interleukin‐1β and interleukin‐18 through their cleavage by caspase‐1. Recognition of mature interleukin‐1β and interleukin‐18 by their receptors has pleiotropic actions, including: (a) recruitment of neutrophils and other innate immune cells, (b) activation of B cells and antibody production and (c) differentiation of T cells. Additionally, activation of the inflammasome induces pyroptosis in immune cells. Pyroptosis occurs when the cytoplasmic protein, gasdermin D, is cleaved by caspase‐1, inducing the N‐terminal gasdermin D fragment to oligomerize and insert into the plasma membrane forming pores. N‐terminal gasdermin D pore formation causes cell lysis and the release of intracellular components (DAMPS) into the intracellular milieu, perpetuating inflammation.

Although insufficient inflammation can lead to persistent infection of pathogens, excessive inflammation can cause chronic or systemic inflammatory diseases. Therefore, it is important that the host balances inflammasome activation. Because inflammasome activation is highly inflammatory, it is tightly regulated to prevent aberrant activation. With the exception of human monocytes, inflammasome activation is a two‐step process; the cell must respond to two sequential signals in order for inflammasome formation and activation to occur (Figure 2). The first step is cell priming, which serves two purposes. The first purpose of priming is the transcriptional and translational upregulation of inflammasome components, including the sensing PRR, caspase‐1 and interleukin‐1β. The second purpose of priming is the post‐translational modification of the PRR and adaptor molecule, ASC. Inflammasome priming occurs through recognition of various PAMPs or DAMPS that engage a subset of multiple toll‐like receptors, NOD1 or NOD2, or by the cytokines, tumor necrosis factor and interleukin‐1β, that lead to transcriptional upregulation of inflammasome components mostly through the activation of the transcription factor nuclear factor‐κB (Figure 2). Importantly, most aspects of inflammasome priming are not specific to the inflammasome being activated. Priming leaves the cells poised to respond to a second signal (step 2). The second step is recognizing a PAMP or DAMP specific to each inflammasome, which then induces inflammasome formation and activation.

Inflammasome formation and activation are initiated through PRR recognition of a PAMP or DAMP specific for the PRR, inducing oligomerization of the PRR (Figure 2). This PRR oligomerization then sets into motion the continued assembly of the inflammasome by the PRR binding to the adapter ASC followed by caspase‐1 binding to ASC. All inflammasome‐inducing PRRs, ASC and caspase‐1 are multidomain‐containing proteins, whose domains are important to the assembly of the inflammasome. Inflammasome assembly occurs by protein‐protein interactions at homotypic (structurally similar) domains. For instance, protein‐protein interactions between many PRRs and ASC occur at pyrin domains found in both proteins, inducing the oligomerization of ASC. Additionally, ASC and caspase‐1 bind at their respective caspase activation and recruitment domain (CARD). Binding of caspase‐1 to the CARD of ASC induces caspase‐1 to oligomerize and autoactivate through self‐cleavage.

It is still not clear how many sensors are capable of forming inflammasomes, with strong literature support for over 10 different inflammasomes, including NLRP1, NLRP3, NLRP6, NLRP12, pyrin, NAIP/NLRC4, RIG‐I AIM2, IFI16, NLRC3, NLP6, recently reviewed elsewhere.^3^, ^4^, ^5^ Here we will focus on discussing the clinical and preclinical data supporting a potential role of NRRP1, NLRP3, pyrin, AIM2 and IFI16 in the context of periodontal disease pathogenesis. Additionally, we will discuss the role of the cytokines matured during inflammasome activation, interleukin‐1β and interleukin‐18. Lastly, we will review the potential of interfering with inflammasome activation and its cytokines for therapeutic purposes.

## 2 NLR INFLAMMASOMES

In the early 2000s, Ting and collaborators^6^ recognized that a handful of related immune regulatory proteins belonged to a large family of mostly uncharacterized proteins recognized by the presence of nucleotide‐binding domains and leucine‐rich repeats.^6^ This led to a standardization of the nomenclature, with the agreement of the PRR family name, NLR (formerly CATERPILLARs, NODs and NACHT‐leucine‐rich repeats).^7^ NLRs are a large family of intracellular proteins that either positively or negatively regulate innate immune responses.^7^ NLRs are found in all animal species and have structural similarity to disease‐resistant proteins in plants. The human genome encodes 23 NLRs, whereas the mouse genome encodes 34. With few exceptions, NLR proteins have tripartite domain organization, including a variable N‐terminal domain, the central nucleotide‐binding domain (also called NACHT) and C‐terminal leucine‐rich repeats. They are divided into four subfamilies dependent upon their N‐terminal domains. These four N‐terminal domains include: (a) acidic transactivator domain, (b) baculoviral inhibition of apoptosis protein repeat (BIR)‐like domain, (c) CARD and (d) pyrin domain.

### 2.1 NLRP1 INFLAMMASOME

Although NLRP1 was the first inflammasome described, exactly how it is activated is still unclear.^1^, ^8^ Although the human genome encodes for a single NLRP1 protein, mice express eight paralogs, *Nlrp1a‐f* and *Nlrp1b2*.^9^ Additionally, murine Nlrp1b has five different alleles that respond differentially to stimuli. The human NLRP1 domain structure deviates from the typical tripartite domain structure and contains an N‐terminal pyrin domain, followed by a nucleotide‐binding domain, leucine‐rich repeats, function to find domain (FIND) and C‐terminal CARD. The mouse domain structure lacks the N‐terminal pyrin domain. Proteolytic cleavage in FIND must occur for NLRP1 to recognize its stimulus, although both portions remain associated. Anthrax lethal toxin is sensed by multiple murine Nlrp1b alleles. This toxin is a protease and N‐terminally processes Nlrp1b, leading to its activation in mice. Although human NLRP1 is neither cleaved nor activated by lethal toxin, experimental cleavage of its N‐terminal sequence is sufficient to activate NLRP1.^10^ This suggests that human NLRP1 may detect pathogen infection by an as yet unknown protease. *Toxoplasma gondii* activates some alleles of rat Nlrp1b. Although polymorphisms in human NLRP1 are linked to congenital toxoplasmosis, there is no evidence that *T. gondii* activates NLRP1 inflammasome.^11^ Muramyl dipeptide has been proposed as the ligand for human NLRP1, although this remains controversial and unsubstantiated.

### 2.2 NLRP3 INFLAMMASOME

The NLRP3 inflammasome responds to structurally and chemically diverse stimuli, including pathogen infections, tissue damage and metabolic changes. Thus, it has been shown to contribute to a substantial number of inflammatory diseases, including diabetes mellitus, obesity and atherosclerosis. The NLRP3 inflammasome is the most highly studied of all the inflammasomes.

Although NLRP3 is activated during a variety of infections and inflammatory diseases, no direct agonist for NLRP3 has been found. Instead, NLRP3 recognizes cell stress in a currently unknown manner. Cellular stressors that are able to activate the NLRP3 inflammasome induce multiple upstream signaling events that are critical for NLRP3 activation. These signaling events include: (a) K^+^, Ca^2+^ and Cl^−^ ion fluxes, (b) lysosomal disruption, (c) mitochondrial damage or dysfunction, (d) production of reactive oxygen species, (e) release of oxidized‐mitochondrial DNA (ox‐mtDNA) and (f) metabolic changes. Although many of these signaling events are not mutually exclusive, it is not clear if they occur in single or multiple signal pathways that then converge upon the activation of NLRP3. Most of these stressors converge on mtROS and ox‐mtDNA, but it is still unclear whether ox‐mtDNA is the direct ligand for NLRP3. Additionally, mitochondrial antiviral signaling protein, an adaptor protein in RNA sensing pathways, has been shown to be important for NLRP3 inflammasome activation during infections by several different RNA viruses and after stimulation with the synthetic RNA poly I:C.^12^, ^13^, ^14^, ^15^ The location of the mitochondrial antiviral signaling protein in the mitochondrial outer membrane protein substantiates a role for NLRP3 sensing of mitochondrial perturbations.

**Figure 3:**
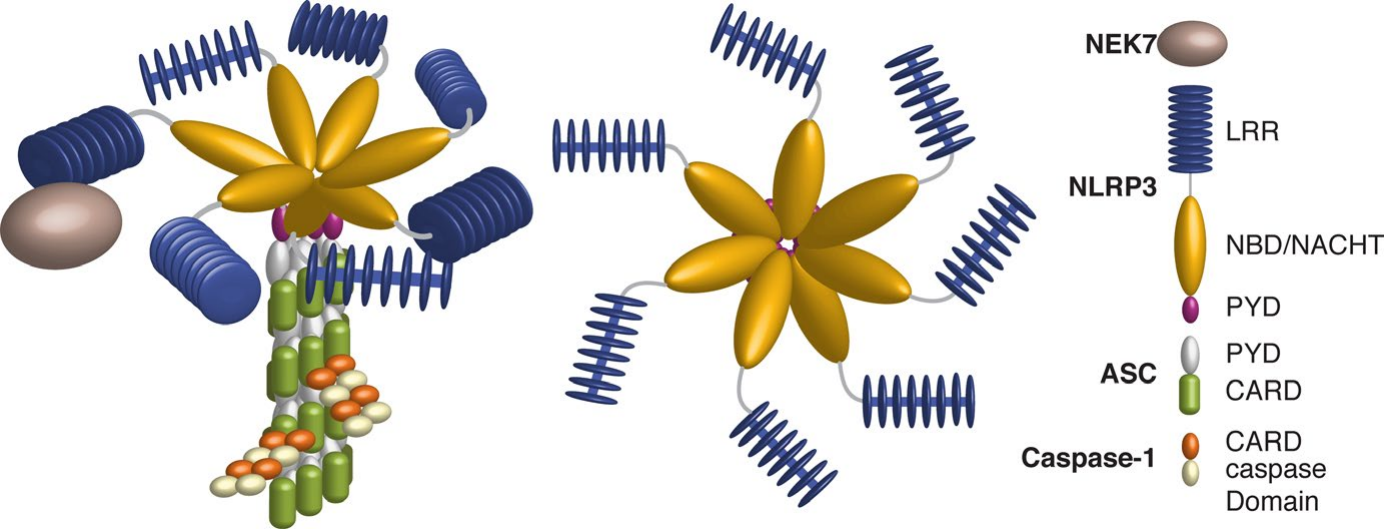
Model of NLRP3 inflammasome. After priming, NLRP3 oligomerizes upon sensing cell stress. NEK7 binds to the NLRP3 leucine‐rich repeat stabilizing the NLRP3 oligomer, which forms a platform of pyrin domains that induces ASC filament formation via pyrin domain‐pyrin domain interactions. Multiple ASC molecules promote caspase‐1 binding and filament assembly through CARD‐CARD interactions. Abbreviations: NEK7, NIMA‐related kinase 7; LRR, leucine‐rich repeat; NBD, nucleotide‐binding domain; PYD, pyrin domain; CARD, caspase activation and recruitment domain

The NLRP3 inflammasome has an additional protein that is unique to it and not found to be associated with other inflammasomes. It was recently recognized that NIMA‐related kinase 7 (NEK7), a serine‐threonine kinase known to be involved in mitosis, is also essential for NLRP3 inflammasome activation.^16^, ^17^, ^18^ The upstream signaling events that induce inflammasome activation also induce NEK7‐NLRP3 interaction. Upon sensing cellular stress, NLRP3 oligomerizes at its nucleotide‐binding domains in a helical manner (Figure 3). NLRP3 oligomerization clusters the pyrin domain of NLRP3, inducing pyrin domain‐pyrin domain‐mediated ASC polymerization. It is not clear whether NEK7 binding occurs prior to NLRP3 oligomerization or after, although recent evidence suggests that it is critical for ASC polymerization.^19^

Activation of the NLRP3 inflammasome is further regulated by post‐translational modifications of NLRP3 and ASC. Multiple post‐translational modifications of NLRP3 occur that are necessary for NLRP3 inflammasome activation, including its phosphorylation, ubiquitination and sumoylation. Additionally, phosphorylation of ASC also occurs and is a necessary step for the activation of the NLRP3 inflammasome.^20^ Thus, the appropriate post‐translational modifications of NLRP3 with the phosphorylation of ASC act to fine tune the NLRP3 inflammasome response and prevent aberrant activation.

### 2.3 NOD1 AND NOD2

NOD1 and NOD2 are CARD‐containing members of the NLR family. NOD1 is ubiquitously expressed, whereas NOD2 is found in myeloid cells, as well as epithelial cells and osteoblasts. NOD1 and NOD2 both recognize different bacterial peptidoglycan components. NOD1 recognizes γ d‐glutamyl‐meso‐diaminopimelic acid (iE‐DAP), whereas NOD2 recognizes muramyl dipeptide, both leading to the activation of nuclear factor‐κB.^21^, ^22^, ^23^ In addition to the well‐known peptidoglycan sensing, NOD receptors were recognized for perceiving perturbations of cellular processes, such as regulation of the actin cytoskeleton and maintenance of endoplasmic reticulum homeostasis.^24^ Although NOD2 does not activate its own inflammasome, activation of NOD2 has been shown to promote activation of both NLRP3 and NLRP1 inflammasomes, both dependent upon muramyl dipeptide recognition.^25^, ^26^ Because activation of NOD1 and NOD2 through recognition of peptidoglycan components leads to nuclear factor‐κB activation, which is necessary for priming the inflammasome, it may be that enhancement of inflammasomes by NOD1 and NOD2 is at least partially due to increased priming effects. Additional pathogen‐sensing mechanisms involving actin cytoskeletal dynamics are attributed to NOD1 and NOD2, although it is not clear if these contribute to enhanced NLRP1 and NLRP3 inflammasome activation.

## 3 ALR

The ALRs are a family of DNA‐binding proteins that contain pyrin domains and DNA‐binding domains (hematopoietic expression, interferon‐inducible nature and nuclear localization [HIN200 or HIN]). They are also referred to as the PYHIN (PYD HIN200) family, based on their domain structure. Humans express four ALRs (AIM2, IFI16, PYHIN1 and MNDA), whereas mice express 13. ALRs are activated through binding DNA at their HIN domains. Of the four human ALRs, only AIM2 and IFI16 form inflammasomes.

ALRs function in both the nucleus and the cytoplasm, where they detect nuclear double‐stranded DNA breaks and sense cytoplasmic DNA. Cytoplasmic DNA is detected by two major sensing pathways leading to different outcomes. The first is the GMP‐AMP (cGAMP)‐synthase (cGAS)‐STING pathway that leads to the production of type I interferons. The second is the activation of inflammasomes. ALRs have been shown to function in both pathways, which leads to type I interferons and interleukin‐1β secretion. Additionally, there is cross‐talk between the cGAS‐STING and inflammasome pathways. ALRs are upregulated by type I interferons induced during activation of the cGAS‐STING pathway (inflammasome priming). Furthermore, activation of cGAS produces the second‐messenger cGAMP, which binds to STING for cGAS‐STING activation, but also enhances activation of the AIM2 inflammasome.^27^

### 3.1 AIM2 INFLAMMASOME

**Figure 4:**
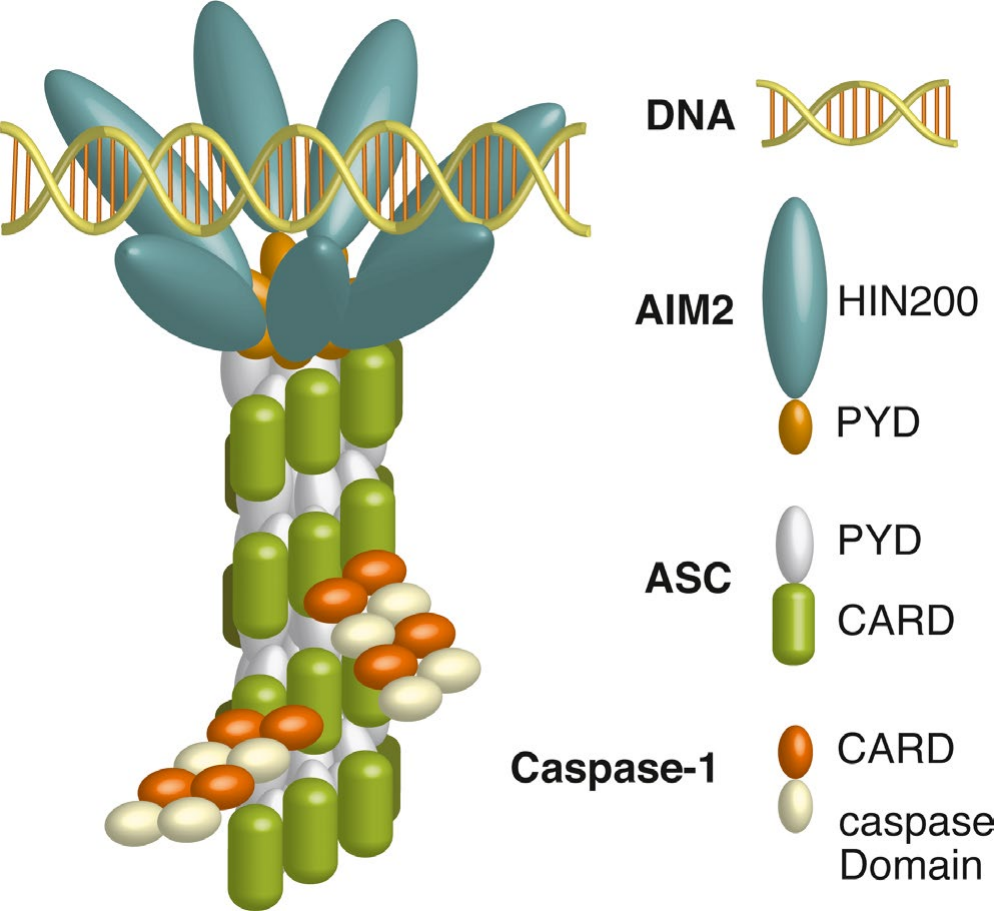
Model of AIM2 inflammasome after assembly. After priming, AIM2 oligomerizes upon binding to DNA through its HIN200 domain and forms a platform of pyrin domains that induces ASC filament formation via pyrin domain‐pyrin domain interactions. Multiple ASC molecules promote caspase‐1 binding and filament assembly through CARD‐CARD interactions. Abbreviations: HIN200, hematopoietic expression, interferon‐inducible nature and nuclear localization; LRR, leucine‐rich repeat; NBD, nucleotide‐binding domain; PYD, pyrin domain; CARD, caspase activation and recruitment domain

Most of the literature on the AIM2 inflammasome centers on it being a protective mechanism during bacterial or viral infections, including *Fransicella tularensis*, vaccinia virus and *Listeria monocytogenes*.^3^ AIM2 resides in an inactive state, with the HIN200 domain folded over the pyrin domain, thus preventing its inadvertent activation. Double‐stranded cytoplasmic DNA from bacterial, viral or self‐DNA binds to the AIM2 HIN200 domain, releasing the HIN200‐pyrin domain association. Multiple AIM2 proteins bind to a single double‐stranded DNA, resulting in the oligomerization of AIM2. This DNA‐induced oligomerization causes clustering of the pyrin domain, which through self‐pyrin domain‐pyrin domain interactions stabilize the assembly and allows for ASC oligomerization and caspase‐1 activation as in other inflammasomes (Figure 4). In addition to its role in inflammasome activation, AIM2 was initially found to be a tumor suppressor and induce cell cycle arrest, and also found to regulate colon cancer tumorigenesis through suppression of AKT, a regulator of cellular proliferation.^28^, ^29^, ^30^

### 3.2 IFI16 INFLAMMASOME

Although most inflammasomes form in the cytosol, IFI16 is the only sensor that has been reported to form an inflammasome in the nucleus. Nuclear IFI16 binds to episomal viral DNA genomes and induces an inflammasome, specifically in response to Listeria monocytogens, herpesvirus and lentivirus infections.^31^, ^32^, ^33^ Structurally, IFI16 has two HIN200 domains, HINa and HINb, which are connected via a linker region. In addition to double‐stranded DNA, IFI16 also binds single‐stranded DNA via the HINa domain, and preferentially binds quadraplex DNA structures that are intrinsic to many viral genomes and GC‐rich DNA.^34^ Although IFI16 is predominately located in the nucleus, it can shuttle between the nucleus and the cytoplasm, where it has other functions.^32^ Although IFI16 has additional DNA sensing roles in the cytoplasm, there is no evidence that it forms an inflammasome from detection of cytosolic DNA.

IFI16 is a multifunctional protein due to its ability to bind to various target proteins and, in turn, modulate a variety of cell functions, including cell cycle regulation, apoptosis/pyroptosis, DNA damage responses and inflammation.^35^, ^36^ Although there is not a murine homolog for IFI16, murine Ifi204 and Ifi205 have shown similar structures and functions in innate immune responses, cell differentiation and proliferation.^37^, ^38^, ^39^, ^40^, ^41^ Differing from the human ALRs, however, the murine homologs do not function in the cGAS‐STING pathway.^42^

Among the different functions it can perform, IFI16 is shown to have inflammasome antagonizing functions. Although it can promote AIM2 inflammasome formation by upregulating AIM2 expression during priming, it is also shown to antagonize the AIM2 inflammasome.^43^ IFI16 lacks the N‐terminal pyrin domain and it can bind to the same DNA as AIM2 and inhibit AIM2 inflammasome formation. IFI16 is also shown to heterodimerize with AIM2 and, therefore, modulate inflammation.^43^, ^44^, ^45^, ^46^ Thus, IFI16 has multiple roles related to inflammasomes that are dependent upon the protein binding, location and splice variation.

## 4 PYRIN INFLAMMASOME

Pyrin was first recognized as the initiator of familial Mediterranean fever, the most common monogenic autoinflammatory disease.^47^, ^48^ These mutations in familial Mediterranean fever lessen the threshold of activation for the pyrin inflammasome, translating into a hyperinflammatory response.^49^, ^50^ Pyrin is unique among PRRs in that it does not fit into any of the known PRR families. It is the protein for which the pyrin domain was named. It has an N‐terminal pyrin domain, then a long linker, followed by a B‐box, a coil‐coil domain and finally a B30.2 at the C‐terminus.^51^ The pyrin inflammasome recognizes inhibition of the Rho family of GTPases. The RhoA family regulates actin cytoskeletal dynamics that many bacterial pathogens modify in order to invade host cells for their survival. Pyrin does not directly sense modified RhoA, but rather senses impairment of RhoA activity from RhoA modifications, sequestration of RhoA or stimulation of its GTPase activity.^51^ Like the other inflammasomes described here, activation of pyrin recruits the linker ASC binding through pyrin domain‐pyrin domain binding. ASC then oligomerizes, followed by caspase‐1 recruitment and activation.

## 5 INFLAMMASOMES AND DISEASE PATHOGENESIS

The same innate immune signaling receptors that are critical for protective anti‐infectious immune responses are also the mediators of the heightened inflammatory state that is found in several noncommunicable diseases.^52^ Therefore, multiple studies have explored inflammasomes in the context of several inflammatory, autoinflammatory and autoimmune diseases.^5^, ^53^ Inflammasomes are primed and activated by PAMPs and, therefore, involved with infection. However, the presence/accumulation of sterile danger signals (DAMPs) in tissues that occurs in many common diseases that present later in life also upregulates inflammasome proteins in diseased tissues. This suggests that the priming step of inflammasome activation has occurred during infection and disease, with a potential for further inflammasome activation and inflammatory cytokine secretion.

Genetic alterations of inflammasome components and increased tissue expression of inflammasome‐related proteins are clinically correlated with several disease phenotypes.^4^, ^5^, ^54^ In this section we will discuss the current evidence from clinical and preclinical studies supporting a role for inflammasomes in some of these conditions, including periodontitis. The diseases discussed in this section were selected based on reports of clinical disease comorbidity with periodontal disease and the current evidence supporting inflammasome involvement. Clinical comorbidity can suggest similar underlying biological principals of different entities that predispose patients for the development of different conditions. We will not discuss data from studies reporting only in vitro results. We have included only selected candidate gene studies due to the false‐positive results and reporting bias that can occur with these studies.

Before we start, it is important to clarify that autoinflammatory diseases differ from autoimmune diseases, in which the first is the innate immune system directly causing tissue inflammation and the latter is the adaptive immune system that directly causes the damage.^53^ Autoinflammatory disorders were first recognized nearly 20 years ago as distinct immunological entities in which there is an innate immune dysregulation that lacks high titers of autoantibodies and self‐reactive T cells.^54^ The hereditary periodic fever syndromes were the first described examples of autoinflammatory diseases and comprise a group of rare, multisystem disorders characterized by recurrent episodes of fever in association with inflammation that affects many tissues.^54^ Skin and mucosal inflammation is a common feature of many autoinflammatory diseases. These syndromes result from different genetic alterations and include familial Mediterranean fever, Crohn's disease and Behcet disease, among many others.^54^

### 5.1 NOD1 AND NOD2

NOD receptors have been included in this section because, in addition to microbial and damage sensing, NOD receptors also promote activation of NLRP3 and NLRP1 inflammasomes.^24^, ^25^, ^26^ The importance of NOD2 in inflammatory diseases is most strongly supported by the association of mutations in NOD2 and the increased risk for developing Crohn's disease, an autoinflammatory disorder of the gastrointestinal tract. The most common polymorphisms associated with Crohn's disease are located in the leucine‐rich repeat domain of NOD2 and include R702W, G908R and L1007fsinsC.^24^, ^55^, ^56^ Individuals with these variants have an increased risk for developing Crohn's disease. Although the exact mechanisms by which these mutations lead to disease is still not clear; data indicate that these mutations impair the mucosal barrier function due to a deficiency in bacterial clearance and activation of toll‐like receptors and Th1 immune responses.^54^, ^57^ Recent studies suggest that these defects may also alter the recognition of endoplasmic reticulum stress‐induced NOD1/NOD2 activation and further contribute to the development of Crohn's disease.^24^, ^54^

Crohn's disease and ulcerative colitis are classified under the umbrella of inflammatory bowel diseases. Although the pathogeneses are distinct, both diseases have multiple similarities, including the chronic debilitating inflammation of the gastrointestinal tract that is partly driven by defects in the innate immune system in response to commensal gut bacteria. It has been suggested that inflammatory bowel disease and periodontal disease share similar immunopathogenic pathways, in that both entities show tissue‐destructive mucosal inflammation directed against commensal microbiota.^58^ Therefore, we propose that some individuals may have a systemic abnormal defensive inflammasome response that is initiated during the sensing of the commensal microbiota by NOD receptors. A recent meta‐analysis of nine cross‐sectional studies (n = 1297) concluded that inflammatory bowel disease was associated with increased risk of periodontitis (332 more diseased individuals per 1000 individuals; *P* < .001) compared with noninflammatory bowel disease individuals.^59^ Individuals with inflammatory bowel disease (either Crohn's disease or ulcerative colitis) had an average of one tooth less (*P* = .090) than noninflammatory bowel disease individuals. Other oral mucosa manifestations of inflammatory bowel disease reported include aphthous ulcers, cobblestoning (fissures/ulcers in separate islands of mucosa with the appearance reminiscent of cobblestones) and pyostomatitis. These additional findings support an alteration in the epithelial barrier and/or host response in oral mucosal tissues that could be parallel to what is observed in the gut.

There is a paucity of information regarding the relevance of NOD1 and NOD2 in periodontitis. Healthy and diseased human gingival tissues are shown to express high levels of NOD1 and NOD2 in epithelial cells and inflammatory cells, with no difference reported among different periodontal conditions.^60^, ^61^, ^62^ In a preclinical study Nod2^−/−^ and Rip2^−/−^ (downstream kinase of Nod1 and Nod2) mice showed a significant reduction of experimental alveolar bone resorption and osteoclastogenesis, supporting NOD2 as a driver of periodontal bone loss.^63^ This result is contradictory to the findings of a collaborative effort developed by the authors in which Nod2^−/−^ mice showed no difference in the amount of bone loss compared with controls.^64^ This could be attributed to the different types of model used to study experimental periodontitis (injection of heat‐killed *Aggregatibacter actinomycetemcomitans* into murine gingival tissues in their study vs ligature model developed by Jiao et al).^64^ Although the inflammatory infiltrate in the gingival tissues did not seem to be affected by Nod2, the osteoclastogenesis was significantly reduced in mice lacking Nod2 in the gavage model.^63^ We speculate that Nlrp3 inflammasome assembly was affected in Nod2‐ablated mice, as signals originating from the bone matrix can act as DAMPs, activate the NLRP3 inflammasome and promote osteoclast differentiation.^65^ Neither study evaluated the effect of Nod2 in the mucosal barrier function of the gingival tissues, as reported to be the main effect on the development of Crohn's disease.^54^, ^57^ In the ligature model of periodontitis, Nod1 (rather than Nod2) drives the alveolar bone resorption, with decreased bone loss (approximately one‐third less compared with wild‐type), decreased interleukin‐β levels, decreased osteoclast numbers and decreased neutrophil migration observed in mice lacking Nod1 and Ripk2 (a mediator of NOD1 and NOD2 signaling).^64^ In summary, the current data demonstrate potential involvement of NOD1 and NOD2 in the pathogenesis of periodontitis. Additional studies involving NOD receptors, DAMPs and epithelial barrier integrity are needed to clarify further the role of these receptors in periodontal disease.

### 5.2 NLRP1

NLRP1 is the main inflammasome in the skin.^54^ Genetic mutations associated with some autoinflammatory diseases are present in either NLRP1's pyrin domain or leucine‐rich repeats domain and lead to constitutive activation of NLRP1. Although NLRP1 is the founding member of the inflammasome family,^8^ most of the studies that have explored its role have centered on autoinflammatory diseases, with no information on the periodontal status of the individuals, and can be reviewed elsewhere.^54^ It should also be noted that although NLRP1, which has many genetic variants in mice and rats, forms well‐defined inflammasomes in these rodent models, the activation of the human NLRP1 into an inflammasome is less well understood.^5^ A meta‐analysis evaluating a total of 37 candidate gene studies among 37 033 cases and 54 716 controls of 10 genetic variants found NLRP1 rs12150220 (odds ratio = 0.71, 95% CI = 0.55‐0.92, *P* = .01) to be significantly associated with type 1 diabetes.^66^ Because it is known that individuals with type 1 diabetes have increased susceptibility to periodontitis,^67^ it leads to further speculation as to whether the alteration of NLRP1 could partially contribute to the clinical comorbidity in particular individuals. Only one clinical study so far has evaluated NLRP1 in the context of periodontal disease.^68^ NLRP1 was shown to be expressed at very low levels in the gingival tissues of healthy, chronic and aggressive periodontitis, with expression more frequently observed in the epithelium and connective tissue of individuals with aggressive periodontitis (n = 65).^68^ The data indicate that NLRP1 function is still not well defined in periodontal disease.

### 5.3 NLRP3

Being the most studied inflammasome in clinical diseases, NLRP3 has been implicated as having a role in several inflammatory and autoimmune diseases, including atherosclerosis, diabetes mellitus, obesity and rheumatoid arthritis,^4^, ^5^ all of which are diseases known for their clinical association with periodontal disease.^67^, ^69^ The data indicate that in these four conditions there is a dysregulation of the inflammatory response that is partly driven by NLRP3.^5^ In atherosclerosis, the formation of cholesterol crystals that accumulate on arterial walls can become intracellular and appear to lead to vascular inflammation.^70^, ^71^, ^72^ In diabetes mellitus type 2, endogenous and exogenous stimulators of NLRP3 inflammasome have been shown to accumulate in the pancreas, including glucose, islet amyloid polypeptides, reactive oxygen species, neuromodulatory lipids (endocannabinoids) and saturated fatty acids that arise from a high‐fat diet.^73^, ^74^, ^75^, ^76^, ^77^ The accumulation of these stimulators can induce NLRP3 activation and subsequent cytokine expression.^5^ In obesity, NLRP3 and ASC are reported to be upregulated in adipocytes from obese individuals.^78^ Obesity‐associated inflammation leads to functional abnormalities of adipocytes, resulting in elevated circulating levels of free fatty acids in human blood that induces pro‐interleukin‐1β production through toll‐like receptors, providing the first signal (priming) for inflammasome activation.^77^, ^79^ Although the data supporting interleukin‐1β as a driver of rheumatoid arthritis disease pathogenesis are strong,^80^ the data supporting the role of the NLRP3 inflammasome are more limited. In rheumatoid arthritis, individuals with active rheumatoid arthritis have increased expression of NLRP3 and NLRP3‐mediated interleukin‐1β secretion in whole blood cells.^81^ In experimental arthritis, deletion of Nlrp3, caspase‐1 and the interleukin‐1 receptor markedly protects against rheumatoid arthritis‐associated inflammation and cartilage destruction.^82^ Together, the data strongly indicate that NLRP3 inflammasome deregulation is implicated in the pathogenesis of atherosclerosis, diabetes mellitus type 2 and obesity and, to a lesser extent, rheumatoid arthritis.

**Figure 5:**
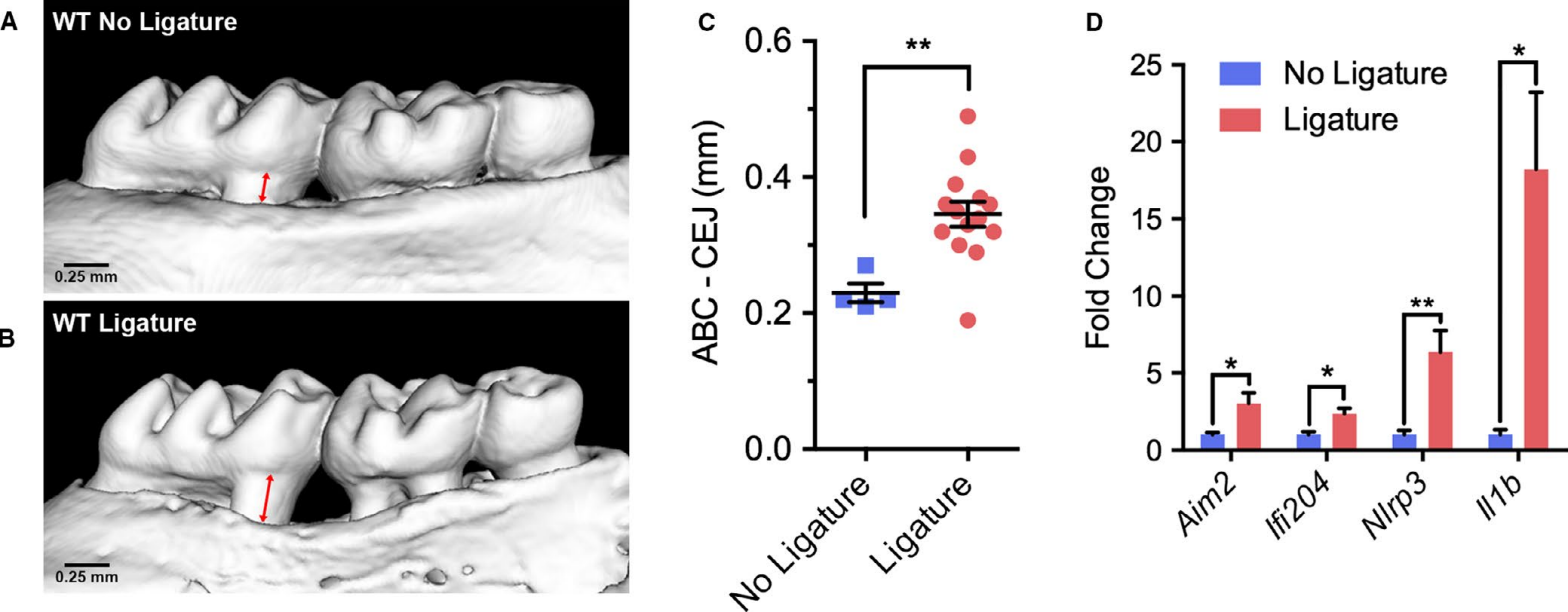
Inflammasome components are increased in experimental periodontitis. Periodontitis was induced in mice via the ligature model for 10 days, as described previously.^173^ Representative images of maxilla micro‐computed tomography showing (A) baseline (no ligature) and (B) alveolar bone loss that developed with disease induction. (C) Alveolar bone quantification shows significant bone loss after 10 days of ligature placement. (D) mRNA expression of inflammasome components are significantly higher in gingival tissues at 10 days postligature when compared with nonligated animals. *P <* *.05, **P <* *.01 as compared with no ligature control

Limited clinical studies have explored the presence of NLRP3 as it relates to periodontal disease. Analyses of human gingival tissue samples showed a significant four‐ to five‐fold increased NLRP3 mRNA expression in chronic periodontitis and approximately a seven‐fold increased expression in aggressive periodontitis when compared with healthy gingival samples.^68^, ^83^ Immunohistochemical results confirmed these findings and showed that the increased NLRP3 expression is more pronounced in the epithelial layer, which could indicate that the epithelium uses NLRP3 to assist the host innate immunity in the resistance to the constant bacterial invasion in the gingival sulcus and tissues.^68^ It appears that even when the inflammation is only present in the gingival tissues and not yet involving bone resorption, NLRP3 levels are already increased (7.7‐fold increased expression in gingivitis gingival samples compared with healthy controls).^83^ Whole saliva samples of individuals with chronic periodontitis and aggressive periodontitis also demonstrate significantly increased levels of NLRP3 compared with healthy individuals, with 3.20 ± 3.12 ng/mL in aggressive periodontitis, 1.56 ± 1.55 ng/mL in chronic periodontitis and 0.530 ± 0.40 ng/mL in saliva from healthy individuals.^84^ Salivary levels of ASC are also reported to have significant differences among individuals with different periodontal status, with 43.62 ± 21.62 pg/mL in healthy individuals, 74.44 ± 63.31 pg/mL in chronic periodontitis and 82.82 ± 73.10 pg/mL in aggressive periodontitis.^84^ Additionally, a positive correlation has been reported between *NLRP3* mRNA levels with *IL1B* and *IL18* mRNA expression levels in gingival tissues.^83^ Data from ligature‐induced periodontitis further support NLRP3 upregulation in experimental periodontitis (Figure 5). Together, the current limited data support the priming of NLRP3 inflammasome components in tissues and saliva samples in the presence of periodontal inflammation.

Evidence also suggests that genetic variants in the *NLRP3* gene are associated with increased risk for developing autoinflammatory Crohn's disease.^85^ A predicted regulatory region on chromosome 1q44 downstream of NLRP3 (rs10733113) was strongly associated with the risk of Crohn's disease (odds ratio = 1.78, confidence interval = 1.47‐2.16) and was consistently replicated in four sample sets from individuals with European descent.^85^ No study so far has reported genetic defects or variants in NLRP3 correlated with periodontal disease.

### 5.4 PYRIN

Mutations in the gene *MEFV* that encodes the protein pyrin leads to familial Mediterranean fever, the prototypic periodic fever syndrome. This group of diseases was the first described examples of autoinflammatory disease and initiated the path to a deeper understanding of the mediation of proinflammatory cytokine interleukin‐1β release that later led to the inflammasome discovery.^54^ Individuals with familial Mediterranean fever experience recurrent episodes of fever with inflammation of serosal membranes in the abdomen, heart and lungs. As the name suggests, this disease is most prevalent in Mediterranean populations, with a relatively high prevalence in Turkey (1:1000).^86^ Approximately one‐third of people with clinical symptoms of familial Mediterranean fever have only one identified mutation in *MEFV*, despite extensive searches for a second mutation.^87^ Five (M694V, V726A, M680I, M694I and E148Q) out of 68 acknowledged MEFV mutations have been reported to be the most common.^88^, ^89^ The spatial arrangement and relocalization of pyrin and NLRP3 inflammasome components during activation are driven by microtubulin dynamics.^90^, ^91^ Colchicine is a highly effective and specific treatment for familial Mediterranean fever and NLRP3 inflammasome that works by binding to tubulin, preventing microtubule polymerization. Additionally, colchicine activates RhoA and suppresses pyrin inflammasome activation.^54^, ^92^

Few studies have been conducted evaluating the periodontal condition of individuals with familial Mediterranean fever. The type of classification selected for familial Mediterranean fever is reported to affect the periodontal clinical findings.^93^, ^94^, ^95^ Individuals with familial Mediterranean fever (n = 81) were shown to have significantly higher clinical measures of periodontal disease severity compared with systemically healthy controls (n = 85), although the clinical magnitude of difference was small (mean, SD probing depth in systemically healthy controls was 2.73 ± 0.86 vs 3.00 ± 0.93 mm in familial Mediterranean fever, *P* = .044; mean clinical attachment level in systemically healthy controls was 2.96 ± 1.10 vs 3.15 ± 1.22 mm in familial Mediterranean fever, *P* = .032). However, several salivary oxidative stress parameters were significantly higher in individuals with familial Mediterranean fever compared with systemically healthy controls (up to six times higher, mean, SD 8OHdG in healthy controls was 12.78 ± 19.88 pg/mL vs 82.80 ± 82.09 pg/mL in familial Mediterranean fever, *P* = .001).^93^ This suggests that periodontal disease in individuals with familial Mediterranean fever may have oxidative stress regulation as a stronger underlying biological driver when compared with systemically healthy individuals. It is also possible that particular genetic mutations of *MEFV* may be more detrimental to the oral microflora environment. Of all the *MEFV* gene mutations, individuals with the M694V mutation showed a higher prevalence of severe familial Mediterranean fever development with early emergence, frequent attacks, need for treatment with higher colchicine doses and frequent amyloidosis occurrence in untreated patients.^96^, ^97^, ^98^ Interestingly, individuals with familial Mediterranean fever with this same pyrin mutation, M694V, were reported to be ~3.5 times more likely to present with periodontitis than individuals with other pyrin mutations.^96^, ^99^ These studies provide clinical indications that pyrin proteins are probably important in the pathogenesis of periodontal disease. No preclinical studies using *Mefv* ^−/−^ mice were identified in the periodontal field.

### 5.5 AIM2 AND IFI16

The ALR family contains four human proteins, of which IFI16 and AIM2 are the only inflammasome‐forming members. Although AIM2 binds to double‐stranded DNA, IFI16 binds to both single‐stranded and double‐stranded DNA encountered during infection by different viruses and intracellular bacteria.^37^, ^100^ Both have additional functions in the detection of DNA beyond their role in inflammasome activation. Importantly, some of IFI16's additional functions include modulation of the AIM2 inflammasome.^44^, ^45^, ^101^

Increased expression of AIM2 has been reported in a number of inflammatory diseases, including inflammatory bowel disease and the skin conditions of psoriasis, atopic dermatitis and venous ulcers.^102^, ^103^, ^104^ In the skin, AIM2 upregulation is seen at sites of individuals with acute and chronic skin barrier disruption‐related inflammation, which can be as striking as more than a 300‐fold increase.^103^ AIM2 priming/upregulation upon skin barrier disruption serves as a first line of defense against invading pathogens. This is beneficial during wound healing, which is usually temporary in the absence of disease. However, the prolonged barrier disruption with AIM2‐interleukin‐1β secretion may contribute to the chronicity of inflammatory skin lesions.^103^ Although less explored in other inflammatory diseases, IFI16 also shows higher expression in inflammatory bowel disease, including Crohn's disease and ulcerative colitis. In active inflammatory bowel disease, a significant two‐fold higher expression of AIM2 and IFI16 is observed in the intestinal mucosa compared with controls,^104^, ^105^ indicating priming/upregulation of these proteins in certain conditions involving barrier function integrity.

**Figure 6:**
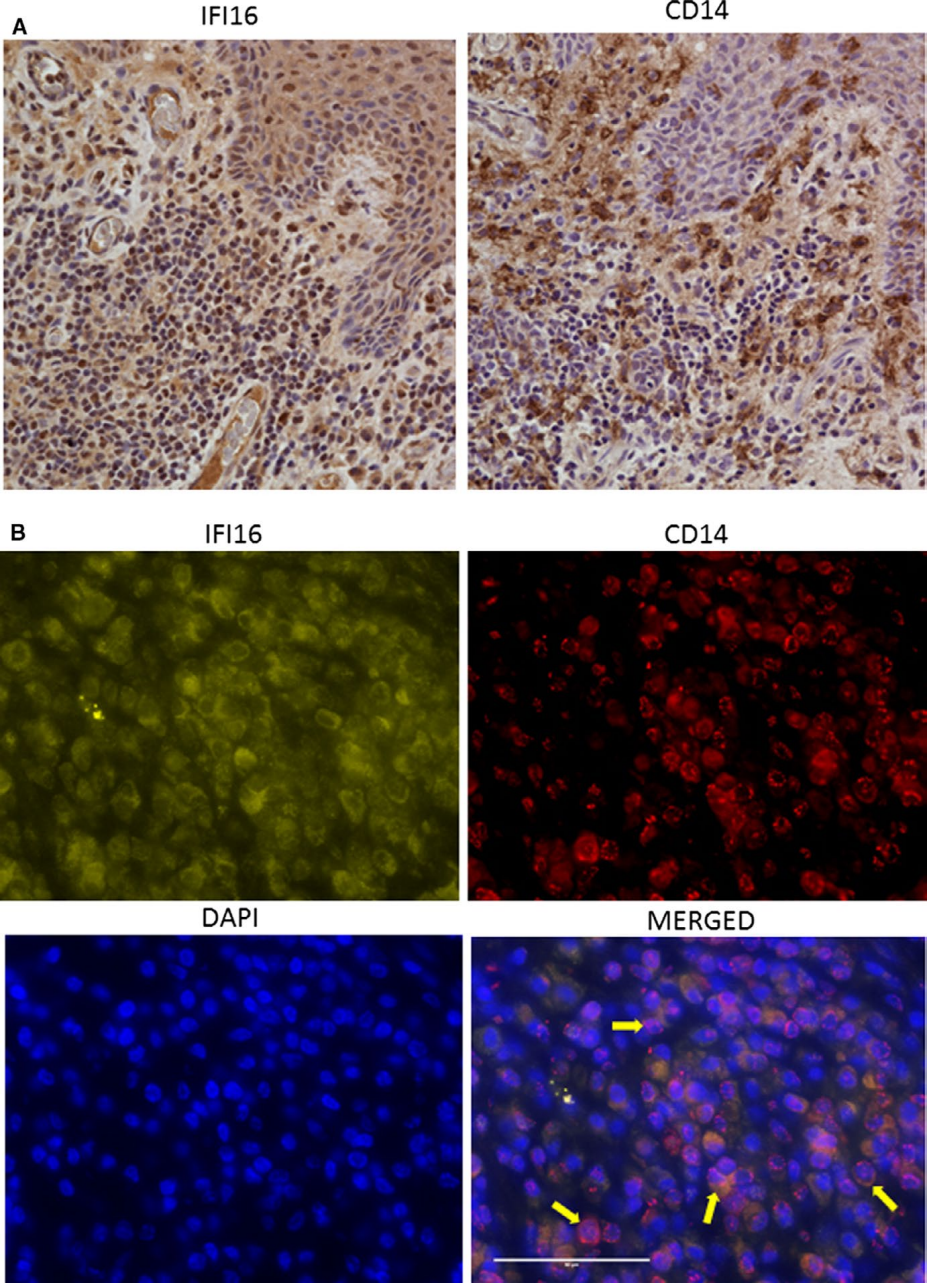
Expression of IFI16 and CD14 in human gingival tissues derived from an individual with chronic periodontitis. A, Immunohistochemistry of IFI16 and CD14 in the inflammatory infiltrate; B, colocalization of IFI16 and CD14 with DAPI for nuclei staining. Yellow arrows show representative cells expressing CD14 and IFI16

There are limited data exploring the role of IFI16 and AIM2 in periodontal disease pathogenesis. Current data report differences in tissue expression based on periodontal disease status, limited tissue characterization and correlation of specific gene variants with periodontitis.^106^, ^107^ In human gingival tissues, AIM2 levels were approximately two‐fold higher in the lamina propria and epithelium of chronic periodontitis compared with healthy controls and aggressive periodontitis.^68^ In that study, AIM2 was mainly expressed in epithelial cells. Previously we have shown that IFI16 and AIM2 are expressed in multiple cells of human gingival tissues, with a homogeneous distribution in the epithelial layer.^106^ In the connective tissue, inflammatory cells and endothelial cells show expression of both IFI16 and AIM2.^106^ Further characterization by our group shows that IFI16 expression in the gingival inflammatory infiltrate coincides with CD14^+^ monocytes/macrophages (Figure 6). In addition, gingival tissues derived from ligature‐induced periodontitis shows a significant two‐fold increase in *AIM2* and *IFI204* expression compared with controls (Figure 5). Together, these studies and preliminary data support a potential inflammasome priming/upregulation of IFI16 and AIM2 in periodontal tissues with disease.

Interestingly, our group has shown that variants in *IFI16* and *AIM2* regions correlate with periodontal disease (genome‐wide association study from n = 4766 European Americans).^106^, ^107^ We further characterized the microbial, biological and periodontal disease clinical parameters of these individuals. Haplotype blocks rs1057028 and rs6940 in the *IFI16* region were significantly correlated with dramatic increases in levels of plaque‐adjusted periodontal microorganisms that ranged from 33% to 275%, with the highest increase associated with plaque levels of *Porphyromonas gingivalis, Tannerella forsythia* and *Campylobacter rectus*. Levels of gingival crevicular fluid‐interleukin‐1β were also significantly higher in individuals with variants on the *IFI16/AIM2* region, ranging from 3% to 5% increases even after plaque adjustment.^106^ Interestingly, individuals with one of the variants, *IFI16* variants (rs6940), are reported to be at a higher risk for developing autoinflammatory Behcet disease, suggesting that alterations in IFI16 may affect the oral mucosal host response.^108^ In addition to genital ulcers, individuals with Behcet disease commonly show oral aphthous ulcers and show increased periodontal clinical parameters of disease.^109^, ^110^, ^111^ Because the recurrent oral ulcers are also known to affect oral hygiene habits, both the bacterial plaque ecology and/or the immune responses to these microorganisms may be affected in Behcet disease. In sum, current literature indicates that inflammasome proteins IFI16 and AIM2 are upregulated in diseased tissues with an epithelial barrier function disruption and *IFI16* and *AIM2* variants that affect the expression or function of these proteins may lead to periodontal disease predisposition and development that may impact the composition of the oral microbiome.

### 5.6 INTERLEUKIN‐1Β

Interleukin‐1 is one of the most studied cytokines in periodontology, having a central role in the host response as a mediator of local tissue destruction and bone resorption.^112^ Both interleukin‐1α and interleukin‐1β are reported to be at least 10 times more potent on a molar basis than tumor necrosis factor‐α, parathyroid hormone or prostaglandin E2 in the induction of bone demineralization.^113^ Clinically, elevated interleukin‐1β levels have been associated with many human diseases In addition, extensive literature supports that gingival crevicular fluid‐interleukin‐1β is associated with different periodontal disease phenotypes. 2, 114, 115 Regardless of the oral condition, the average level of interleukin‐1β in gingival crevicular fluid is reported as 136.8 ± 1.4 (mean, SE) ng/mL, with a high range of variation among individuals.^114^

**Figure 7:**
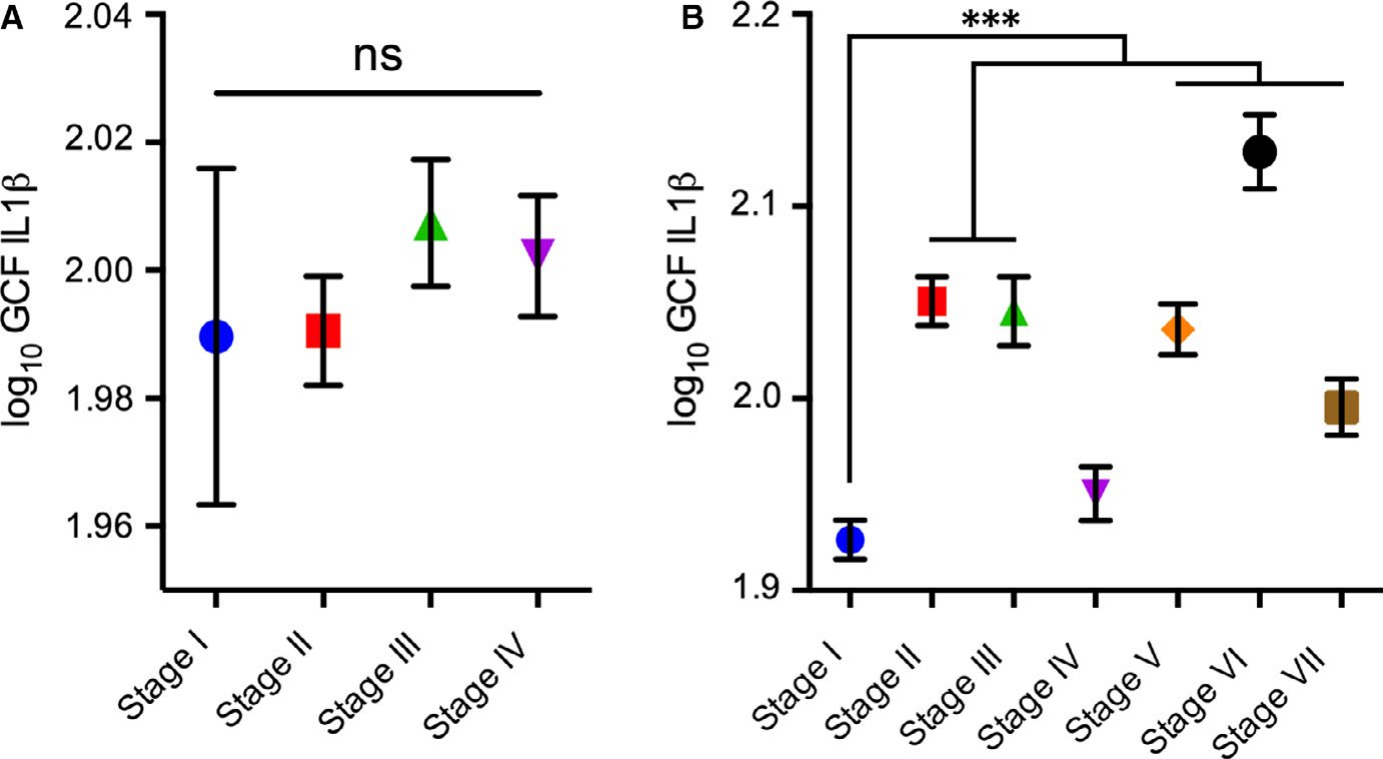
Gingival crevicular fluid‐interleukin‐1β log levels stratified by new periodontal disease classifications in individuals from the Dental Atherosclerosis Risk in Communities (Dental ARIC) study (n = 5809). A, 2017 Classification of Periodontal Conditions^117^, ^118^ B, periodontal profile phenotype.^119^, ^120^, ^121^ Generalized linear models adjusted for age, sex, study center, race, smoking and diabetes history. The data were log‐transformed because they were not normally distributed. WW17 stages were as follows: incipient, moderate, severe and advanced disease; periodontal profile class classification was as follows: stage I –health/incidental disease; stage II – mild disease; stage III – moderate disease; stage IV – severe disease; stage V – mild tooth loss/hi GI; stage VI – moderate tooth loss; stage VII – severe tooth loss. ***P <* *.001 as compared with stage I

Offenbacher and collaborators^114^ have previously evaluated gingival crevicular fluid‐interleukin‐1β levels in 5809 individuals from the Dental Atherosclerosis Risk in Communities study, which is a cross‐sectional study that took place during Visit 4 of the Dental Atherosclerosis Risk in Communities study of community‐dwelling adults aged 45‐64 years conducted in four US communities. In this study, individual average gingival crevicular fluid‐interleukin‐1β was positively correlated to maximum probing depth and bleeding on probing, supporting an association between gingival crevicular fluid‐interleukin‐1β levels and clinical signs of periodontal disease. It is reported that a 22.8‐150 ng/mL concentration range is observed in healthy individuals and 85.8‐882.2 ng/mL in moderate/severe periodontitis.^116^ The 2017 World Workshop on Periodontal Disease Classification (WW17) has proposed a new classification system that involves stages and grades of disease. We evaluated the association of gingival crevicular fluid‐interleukin‐1β levels according to the WW17 classification system^117^, ^118^ and the periodontal profile class stages described in the references.^119^, ^120^, ^121^ No differences were observed in gingival crevicular fluid‐interleukin‐1β levels across the WW17 stages (incipient, moderate, severe and advanced disease), potentially because of the lack of consideration of the grades that requires longitudinal analysis of the individual (Figure 7). The periodontal profile class stages (health/incidental, mild, moderate, severe, mild tooth loss/high gingival inflammation, moderate tooth loss/reduced periodontium and severe tooth loss) showed that individuals in all disease categories were significantly different from stage I (health/incidental disease), which had the lowest concentration (mean, SE 83.75 [1.02] pg/mL), with the exception of stage VII (severe tooth loss; mean, SE 89.84 [1.03] pg/mL; Figure 7). This could be attributed to the fact that most of the diseased teeth of individuals in stage VII (severe tooth loss) have already been lost. Interestingly, individuals in stage VI (severe disease) showed the highest levels of gingival crevicular fluid‐interleukin‐1β (mean, SE 135.25 [1.04] pg/mL). Individuals in this category show mild tooth loss, usually missing six to 10 teeth, and extensive gingival inflammation, but very minimal bleeding on probing. The analysis indicates that high gingival crevicular fluid‐interleukin‐1β is associated with the periodontal disease phenotypes represented by the periodontal profile class stages, but not the phenotypes represented by the WW17 stages.

The influence of genetic control on interleukin‐1β production has been previously supported by twin studies, with an estimated 86% of the variance for interleukin‐1β production being genetically determined.^122^ Among all genetic variations, two *IL1* gene variations (*IL1A* [−889; rs1800587] or the concordant *IL1A* [+4845; rs17561] and *IL1B* [+3954; rs1143634]) are the ones most studied and consistently associated with the progression and severity of periodontitis in Caucasians, with significant associations between the *IL1* variants and periodontitis reported for 19 of 27 studies and validated in two meta‐analyses.^123^, ^124^ A recent study correlated *IL1B* variations with periodontal disease in other non‐Caucasian ethnicities, including African‐Americans, Hispanic and Asians.^125^ Data indicate that the *IL1* gene cluster, including *IL1A*,* IL1B* and *IL1RN* loci, impacts the amount of interleukin‐1β secretion.^126^ It is suggested that the variants cause interleukin‐1 to be secreted at higher levels in gingival tissues, therefore contributing to the development and progression of disease. A recent genome‐wide association study analysis carried out by our group in 4910 European‐American adults identified loci significantly (*P* < 5 × 10^−8^) associated with high gingival crevicular fluid‐interleukin‐1β in the *IL1* gene complex area of chromosome 2.^127^ In this study, 72 single nucleotide polymorphisms were correlated with high gingival crevicular fluid‐interleukin‐1β (upper quartile), including 16 variants in the *IL1B* region (Table 1). *IL1B* [+3954; rs1143634], which is part of the *IL1* genotype previously associated with periodontitis, is among the variants associated with high gingival crevicular fluid‐interleukin‐1β. However, the periodontal phenotype of these individuals was not described in a genome‐wide association study data set. Subjects with high interleukin‐1β levels were also more likely to have severe periodontitis, diabetes, high carotid intima‐media wall thickness, higher body mass index and to be heavier smokers.^127^ To date, variants in the *IL1A‐IL1B* regions have been associated with coronary artery disease,^128^ rheumatoid arthritis^129^ and Behcet disease^130^ in three meta‐analyses of candidate gene studies. Together, this further supports that variants of the *IL1* gene can potentially alter the inflammatory response and predispose for diseases.

**Table 1:** IL1B gene variants significantly associated with high gingival crevicular fluid‐interleukin‐1β (upper quartile)^127^

SNP ID	Chromosome	Closest gene reference	*P*‐value
rs16944	2	*IL1B*	6.53E‐19
rs1143627	2	*IL1B*	6.70E‐19
rs2708916	2	*IL1B*	7.73E‐19
rs10169916	2	*IL1B*	7.76E‐19
rs2708914	2	*IL1B*	8.00E‐19
rs2466446	2	*IL1B*	8.29E‐19
rs13013349	2	*IL1B*	1.79E‐18
rs6735739	2	*IL1B*	5.75E‐18
rs12621220	2	*IL1B*	3.70E‐17
rs1143623	2	*IL1B*	4.91E‐17
rs12053091	2	*IL1B*	7.92E‐17
rs13008855	2	*IL1B*	3.43E‐16
rs11674397	2	*IL1B*	2.62E‐14
rs4334503	2	*IL1B*	2.74E‐14
**rs1143634**	**2**	***IL1B***	**9.94E‐12**
rs7596684	2	*IL1B*	4.08E‐10

Bold represents *IL1B* single nucleotide polymorphisms (SNPs) previously identified as a risk for periodontitis in candidate gene studies.

### 5.7 INTERLEUKIN‐18

Interleukin‐18 belongs to the interleukin‐1 superfamily that was originally discovered as an interferon γ‐inducing factor.^131^ It is secreted by a variety of cell types and strongly augments interferon‐γ production in natural killer cells and Th1 cells.^132^ Although it is also a cytokine matured through inflammasome activation, the role of interleukin‐18 in diseases is significantly less explored as compared with interleukin‐1β.^2^, ^132^ The majority of studies exploring the impact of interleukin‐18 levels in diseases are related to a group of diseases named cryopyrin‐associated periodic syndromes, which are associated with gain‐of‐function mutations in NLRP3 mediated primarily by interleukin‐1β effects.^52^, ^54^, ^133^ There is currently no study reporting periodontal disease parameters and cryopyrin‐associated periodic syndromes.

Interleukin‐18 levels have previously been measured in gingival tissues and saliva samples of individuals with different oral disease status in small clinical studies. Gingival tissues showed no difference in the levels of interleukin‐18 when comparing chronic periodontitis (n = 18), aggressive periodontitis (n = 12) and healthy (n = 9) individuals.^134^ Saliva samples from nonsmoking individuals with chronic periodontitis showed significantly higher levels of interleukin‐18 (275.05 ± 289.46 pg/mL; mean ± SD) when compared with healthy controls (143.71 ± 103.68 pg/mL; mean ± SD).^135^ Candidate gene studies evaluating polymorphisms have not identified single nucleotide polymorphisms in the *IL18* region correlated with periodontal disease.^136^

The current evidence suggests that biological alterations of the inflammasome, either by genetic variations or priming, are correlated with the development of different complex diseases. Inflammasome dysregulation appears to lead to a defective bacterial clearance and impaired epithelial/mucosal barrier function that is translated to disease predisposition. Clinical comorbidity of some inflammasome‐driven entities with periodontal disease further supports this concept. Although previous twin studies suggest that 50% of the variance of periodontal disease has a genetic composition, it is clear that this will not be explained by a single altered protein. A genome‐wide association study and candidate gene studies in type 2 diabetes concluded that all associated variants identified affected the risk of disease by <40%, and most affected risk by closer to 15%.^137^, ^138^, ^139^ As previously suggested, it appears that periodontal disease is actually a group of distinct biological conditions with similar overlapping clinical presentations.^127^ The challenge is to identify these different groups of periodontal disease in order to target individual biological alterations that will lead to improved and tailored therapies.

## 6 THERAPEUTICS TARGETING THE INFLAMMASOME

The host response is well recognized as a major contributor to periodontal tissue damage by supporting a nonresolving inflammation and dysbiosis.^140^ It is acknowledged that most of the biological approaches to improve periodontal treatment have been focusing on direct microbial management (antibiotic treatments) rather than approaches that will modulate the host response.^141^ The inappropriate inflammasome activity with continuous production of proinflammatory cytokines is believed to contribute to the development of many diseases. Therefore, inhibitors/antagonists targeting inflammasome components, activation status and cytokine production are an attractive approach for treating periodontal disease.

It is important to clarify that upregulation (priming) of inflammasome components (NLRP3, AIM2, IFI16, caspase‐1, pro‐interleukin‐1β) in a tissue is only the first step of inflammasome activation (Figure 2). Multiple PAMPs and DAMPs will cause these priming events by binding to their cognate receptors and inducing upregulation. Although this does not imply inflammasome activation, protein overexpression (priming) in diseased tissues is currently used as an indicator for a rationale of therapeutic targeting of inflammasome activation in several diseases.^5^, ^52^

Although most of these inhibitor/antagonist treatments are given via oral/subcutaneous routes, as reaching most inflamed tissues requires systemic processing and access to diseased tissues, the easy access to a periodontal pocket provides the opportunity for direct drug delivery that is potentially possible depending on the chemical composition of the drug. Intrapapillary injections were previously administrated for drugs targeting of interleukin‐1 and tumor necrosis factor activity in experimental periodontitis in primates.^142^ Diseases like osteoarthritis are also being approached by using drugs that are formulated in the form of a gel, such as OLT1177, rather than systemic administration.

In this section we will discuss clinical and preclinical studies evaluating different strategies that target inflammasomes as a therapeutic approach. We will not include pathways involved in priming of inflammasomes, which would include multiple PRRs and signaling pathways. Although there is broad evidence that lipoxins and resolvins broadly decrease inflammation in diseases (including periodontal disease), the data suggest that they mostly affect the inflammasomes at the priming stage^143^, ^144^ and, therefore, will not be discussed in this review. Therapeutic inhibition or suppression of the inflammasome can be targeted by three strategies:

- Inhibiting the upstream intracellular signaling pathways
- Blocking inflammasome components
- Inhibiting inflammasome‐mediated cytokines (interleukin‐1β and interleukin‐18)

### 6.1 Inhibiting the upstream intracellular signaling pathways

There are multiple upstream signals that can lead to NLRP3 inflammasome activation, including K^+^, Ca^2+^ and Cl^−^ flux, lysosomal disruption, mitochondrial damage and release of reactive oxygen species. Most pathways converge on mitochondrial stress and, with the exception of viral activation, also leads to the release of reactive oxygen species. The relevance of oxidative parameters in periodontal disease has been previously proposed, with periodontal disease associated with decreased saliva antioxidants and increased oxidative damage.^145^, ^146^, ^147^ Therefore, we will discuss current trials using drugs that decrease the production of reactive oxygen species and further inflammasome activation. Reactive oxygen species produced in the cell (potentially by mitochondria) are known as direct or indirect activators of the NLRP3 inflammasome.

Allopurinol (Zyloprim/Aloprim®) is one of the drugs discovered in the Burroughs Wellcome program that started in the 1940s that led to the 1988 Nobel Prize in Physiology and Medicine award to Gertrude B. Elion and George H. Hitchings, shared with James W. Black, for “discoveries of important principles for drug treatment”.^148^ Allopurinol is the prototypical xanthine oxidase inhibitor that has been in the market for decades and is considered a standard treatment of hyperuricemia associated with treating gout (a form of arthritis that is NLRP3 inflammasome dependent^149^) and kidney stones.^148^ The drug is being tested for treating individuals with type 2 diabetes and has reached phase III trials after promising results in experimental diabetes murine models (Table 2).

**Table 2:** Drugs inhibiting inflammasome components

Direct inhibitor	Condition tested	Inhibition mechanism	Inflammasome component	Clinical status	ClinicalTrials.Gov	References
Allopurinol (Zyloprim)	Diabetes mellitus (type 2)	Inhibition of xanthine oxidase, reduction of uric acid	Reactive oxygen species	Approved for treatment of gout and kidney stones Diabetic neuropathy phase III testing	NCT02533648 NCT00430248	174
SS‐31 (Elamipretide, Bendavia, MTP‐131)	Heart failure, primary mitochondrial disease, Barth syndrome	Stabilization of cardiolipin (mitochondrial membrane component)	Reactive oxygen species	Approved for treatment of gout and kidney stones; Heart failure phase I testing PMD phase II testing	NCT02814097 NCT02976038 NCT03098797 NCT03323749 NCT02388464	151
Nicotinamide riboside (NR)	Atherosclerosis, diabetes, Coronary artery disease	Activation of SIRT3, blunting of NLRP3 response similar to what occurs during fasting	Reactive oxygen species	Available as dietary supplement; Phase II completed to evaluate effects on immune response	NCT02812238	175

Another antioxidant drug being evaluated in clinical trials is being tested for the treatment of several inflammatory conditions by stabilizing a phospholipid that is exclusively expressed in the inner mitochondrial membrane, named cardiolipin. The therapeutic drug SS‐31 (Elamipretide, Bendavia, MTP‐131) is member of the Szeto‐Schiller (SS) peptides that selectively targets and stabilizes cardiolipin in the inner mitochondrial membrane. This stabilization prevents excessive reactive oxygen species production and preserves the electron carrying function of cytochrome c. The drug currently shows benefits on treating highly complex diseases that share a pathogenesis of bioenergetics failure as mitochondrial dysfunctions result in insufficient energy to maintain cell function (Table 2).^150^, ^151^ No preclinical or clinical studies evaluating these drugs were identified in the periodontal field.

### 6.2 Blocking inflammasome components

#### 6.2.1 Caspase‐1

Caspase‐1 activation precedes interleukin‐1β and interleukin‐18 release after inflammasome activation. Three drugs targeting caspase‐1, Emricasan, VX‐740 (Pralnacasan) and VX‐765, have been tested in humans.^52^, ^152^ The most developed caspase‐1 inhibitor for therapeutic use is VX‐765, a reversible inhibitor of caspase‐1 that is metabolized by plasma esterases into its active form.^153^ VX‐765 has been used for treating psoriasis and epilepsy, reaching phase II clinical trials (Table 3).^154^

**Table 3:** Drugs blocking inflammasome components

Direct inhibitor	Condition tested	Inhibition mechanism	Inflammasome component	Clinical status	ClinicalTrials.Gov	References
VX‐765	Psoriasis, epilepsy	Inhibition of caspase‐1	Caspase‐1	Psoriasis phase II testing completed Alveolar bone loss inhibition in experimental periodontitis	NCT00205465 NCT01048255 NCT01501383	176
Ibrutinib (Imbruvica)	Mantle cell lymphoma, chronic lymphocytic leukemia, Waldenstrom's macroglobulinemia	Inhibitor of Bruton's tyrosine kinase (BTK) (inhibits ASC phosphorylation)	NLRP3	Approved for treatment of B cell cancers Chronic graft‐vs‐host disease phase I testing Alveolar bone loss inhibition in experimental periodontitis	NCT01578707 NCT03604692	156, 157
IDN‐6556 Emricasan	Diabetes, nonalcoholic steatohepatitis	Pan‐caspase inhibitor; prevents excessive apoptosis	Caspase‐1	“Fast‐track” status for development of nonalcoholic steatohepatitis cirrhosis Diabetes phase II tested	NCT01653899 NCT03205345	177
Colchicine (Colcrys)	Gout, familial Mediterranean fever, Behcet's disease	Blockage of tubulin assembly, inhibition of NLRP3 and interleukin‐1β processing and release	NLRP3 and pyrin	Approved for treatment of gout and familial Mediterranean fever	NCT02145589 NCT00506883	159
OLT‐1177 Dapansutrile	Heart failure, osteoarthritis, Schnitzler syndrome	Prevents oligomerization, inhibiting NLRP3‐ASC interaction, and NLRP3‐caspase‐1 interaction	NLRP3	Heart failure phase I testing Phase II for Schnitzler syndrome	NCT03595371 NCT03534297 NCT01768975	178

**Figure 8:**
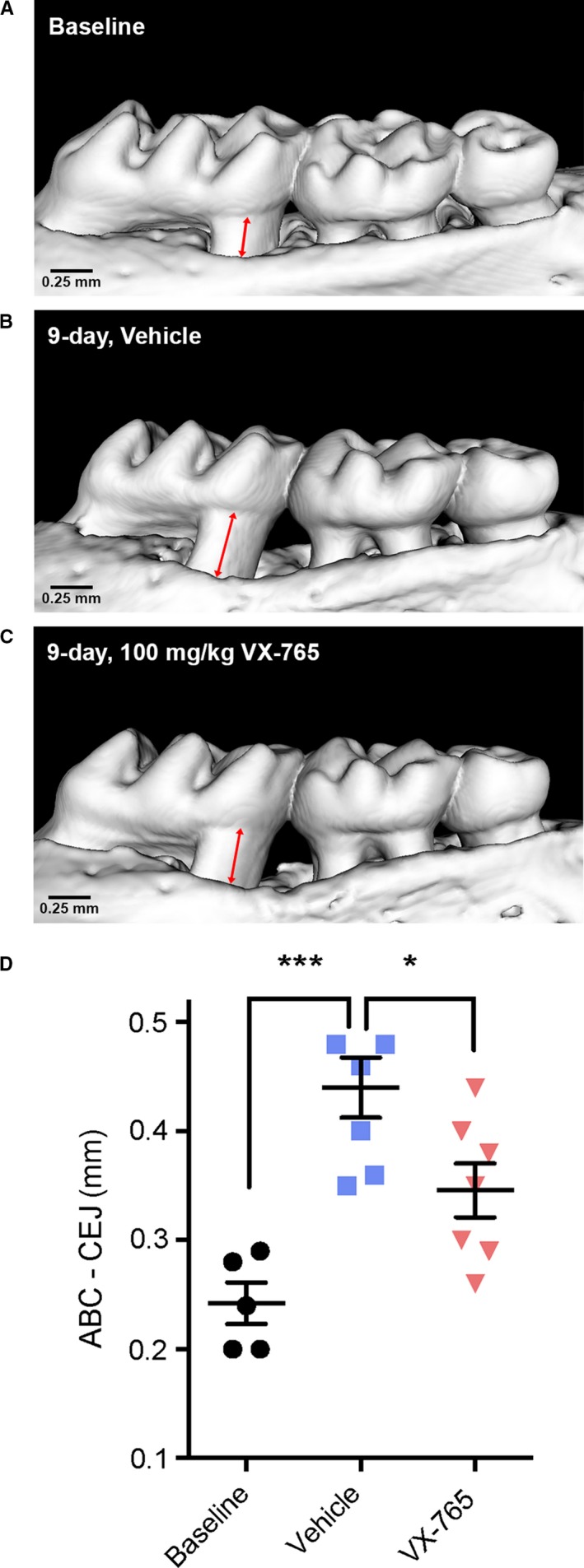
Caspase‐1 inhibition blocks approximately 50% of alveolar bone loss in mice. One day before the ligature placement, wild‐type male mice (n = 5‐7) started receiving a twice a day oral delivery of vehicle (DMSO) or caspase‐1 inhibitor (VX‐765 at 100 mg/kg) for 10 days. After 1 day of drug delivery, mice were induced for experimental periodontitis for 9 days based on a previously described protocol.^155^ A‐C, Mean representative images of each group showing alveolar bone loss at 9 days postligature placement. D, Measurements taken from the alveolar bone crest (ABC) to the cementum‐enamel junction (CEJ) show significant inhibition of alveolar bone loss as compared with vehicle control. *P <* *.05, ***P <* *.001

In order to gain a greater understanding of the effect of caspase‐1 inhibition in the periodontal host response, we administrated the caspase‐1 inhibitor VX‐765 using a protocol previously shown to decrease bone loss in murine osteoarthritis.^155^ Male mice received daily oral dosages of 100 mg/kg of VX‐765 for 11 days, with the drug administration starting 1 day prior to the ligature placement and bone loss was monitored after 10 days. Our micro‐computed tomography results show that caspase‐1 inhibition significantly decreased ~50% of alveolar bone loss that is normally observed in this model of disease (Figure 8). The preliminary result further supports the concept of manipulation of inflammasome components to treat periodontitis.

#### 6.2.2 Bruton's tyrosine kinase

Bruton's tyrosine kinase (BTK) is a nonreceptor tyrosine kinase, most prominently known for its indispensable role during B cell development. During the activation of the NLRP3 inflammasome, BTK has been shown to interact with NLRP3 and ASC.^156^ The interaction of BTK with ASC is crucial for ASC oligomerization during NLRP3 inflammasome activation. Although it is not clear whether BTK directly phosphorylates ASC, the kinase activity of BTK is essential for ASC oligomerization and NLRP3 inflammasome activation. The BTK inhibitor ibrutinib (PCI‐32765, Imbruvica) is a Food and Drug Administration‐approved drug for the treatment of a number of B cell cancers, including mantle cell lymphoma, chronic lymphocytic leukemia/small lymphocytic lymphoma, Waldenström's macroglobulinemia and marginal zone lymphoma (Table 3). It is an orally available, selective inhibitor of BTK that inhibits the signaling of B cell chemotaxis and is mostly known for its antitumor and antimetastatic efficacy. In addition to the treatment of different B cell cancers, it is currently being tested to treat chronic graft‐vs‐host disease (Table 3). Inhibition of BTK by pharmacological or genetic means severely impairs activation of the NLRP3 inflammasome. BTK inhibitor efficiently suppresses infarct volume growth and neurological damage in a brain ischemia/reperfusion model in mice.^156^

Recently, another BTK inhibitor, acalabrutinib (ACP‐196), has been evaluated for its potential to prevent alveolar bone loss in experimental periodontitis.^157^ In this study, mice that received intragingival injections of *P. gingivalis*‐derived lipopolysaccharide were also given intraperitoneal injection of acalabrutinib (0.5 mg/kg on days 1, 4 and 7 postligature placement). The results showed that BTK inhibitor ACP‐196 altered osteoclastogenesis and significantly decreased alveolar bone loss by approximately 50% compared with controls. Although the role of inflammasomes was not explored in this study, it is possible that the effect of BTK initiated upstream by binding to NLRP3 and later affecting osteoclastogenesis.^65^ The study suggests that inhibition of BTK is a plausible treatment for bone loss during periodontal disease development.

#### 6.2.3 NLRP3 and pyrin inflammasomes

NLRP3 and pyrin inflammasomes are proposed to recognize perturbations in cellular homeostasis, which is a unique feature when compared with other well‐characterized inflammasomes that recognize PAMPs and DAMPs directly (like AIM2, IFI16). NLRP3 components are one of the main targets currently being evaluated to treat a range of diseases in clinical trials, including heart failure, osteoarthritis, leukemia, psoriasis, diabetes and nonalcoholic steatohepatitis (Table 3).^52^

Colchicine is a therapeutic agent used to treat diseases for over 3000 years that has been Food and Drug Administration approved to treat gout (a form of arthritis) in the USA since 2009.^158^ Colchicine has been studied for its anti‐inflammatory, antioxidant, antimitotic and bone‐protective effects. The primary mechanism of action of colchicine is tubulin disruption, which blocks microtubule assembly and impacts several innate immune pathways, including NLRP3 and pyrin‐inflammasome activation.^54^, ^159^, ^160^ The spatial arrangement and relocalization of NLRP3 and pyrin inflammasome components during activation is driven by microtubulin dynamics.^90^, ^91^ Colchicine is used in the treatment of the inflammasome‐mediated diseases familial Mediterranean fever^160^, ^161^ and gout,^162^ as well as cardiac disease (Table 3).^163^ The effects of colchicine as a therapeutic agent were recently evaluated in experimental periodontitis.^164^ Preliminary evidence indicates that colchicine inhibited ligature‐induced periodontitis in rats at both dosages tested (intraperitoneal injections, 30 and 100 μg/kg/d) when compared with vehicle control in an 11‐day time course experiment.^164^ Among the cytokines evaluated, gingival tissue interleukin‐1β levels significantly decreased to half during colchicine treatment (~800 pg/mL in periodontitis and ~400 pg/mL at baseline and after providing both dosages of colchicine). The data indicate a general decrease in inflammation via the administration of colchicine that could be, in part, due to NLRP3 and/or pyrin inflammasomes.

### 6.3 Inhibiting inflammasome‐mediated cytokines

#### 6.3.1 Interleukin‐1β

Interleukin‐1β is the most potent proinflammatory cytokine released by activation of inflammasomes and is implicated as the effector molecule in many inflammasome‐driven diseases.^52^, ^165^ Global inhibition of interleukin‐1β is currently used in the clinical setting by either a monoclonal antibody targeting interleukin‐1β (canakinumab), by a modified interleukin‐1β receptor antagonist (anakinra) or by a soluble decoy receptor (rilonacept). These approved drugs are being used to treat rheumatoid arthritis,^166^ atherosclerotic disease^167^ and diabetes,^168^ with promising results (Table 4). Among the therapeutic interventions, the CANTOS trial, with 10 061 individuals, recently demonstrated that individuals receiving 150 mg of canakinumab subcutaneously every 3 months had a significant lower rate of recurrent cardiovascular events than placebo.^167^ It is important to note that these drugs are shown to moderately increase the risk of infection and there are currently no specific prevention strategies being recommended.^169^ It is speculated that further understanding of the inflammasomes will allow targeting of other components more specific to different inflammasomes that could be less immune‐suppressive than a global interleukin‐1 therapy.^52^ However, the CANTOS trials were received with enthusiasm in the medical field, given that many treatments have proven to be unsuccessful in treating coronary disease. It is important to note that canakinumab was associated with a small but statistically significant risk of fatal infection but still had no effect on all‐cause mortality. Of interest, a prespecified analysis demonstrated a reduction in the incidence of lung cancer in the pooled canakinumab population (*P* = .0001 for trend across groups) and a reduction in lung cancer‐related mortality (*P* = .0002). Therefore, the CANTOS trial can be acknowledged as a landmark trial for its bold achievement in translating state of the art biology to the bed side to examine the true importance of inhibiting interleukin‐1β in diseased individuals. The results of the trial may act as a strong stimulus to trial other safer, more widely available, less expensive therapies capable of inhibiting the inflammasome.^170^

**Table 4:** Drugs inhibiting final products of inflammasomes

Direct inhibitor	Condition tested	Inhibition mechanism	Inflammasome component	Clinical status	ClinicalTrials.Gov	References
Canakinumab, ACZ885 (ILARIS)	RA, atherosclerosis	Monoclonal antibody targeting interleukin‐1β	Interleukin‐1β	Approved for treatment of cryopyrin‐associated periodic syndromes and systemic juvenile idiopathic arthritis; Rheumatoid arthritis phase II testing terminated Atherosclerosis phase II completed	NCT00505089	167, 179
Rilonacept IL‐1 trap (Arcalyst)	Cryopyrin‐associated periodic syndromes, familial Mediterranean fever	Binds and neutralizes interleukin‐1	Interleukin‐1	Cryopyrin‐associated periodic syndromes phase II testing completed Familial Mediterranean fever phase II testing completed	NCT00288704 NCT01045772 NCT00582907	180
Anakinra (Kineret)	Rheumatoid arthritis, familial Mediterranean fever	Blocks interleukin‐1 binding via competitive inhibition of interleukin‐1 type I receptors	Interleukin‐1 receptor	Approved for treatment of rheumatoid arthritis Familial Mediterranean fever phase III testing completed	NCT01705756	181
GSK1070806 (anti‐IL‐18)	Diabetes mellitus (type 2), Behcet's disease	Neutralization of interleukin‐18	Downstream Th1 cytokines, including interleukin‐1β; serum interleukin‐18	Diabetes mellitus (type 2, phase II completed, no effect observed) Behcet's disease phase II testing	NCT01035645 NCT03522662 NCT01648153	171

In primate experimental periodontitis, a combined treatment to block interleukin‐1 and tumor necrosis factor activity demonstrated a significant 50% reduction of levels of radiographic bone loss over that found in control sites.^142^ The regimen provided was a three times per week intrapapillary injections of soluble human recombinant interleukin‐1 receptor consisting of the extracellular domain of the type I receptor and a fusion protein consisting of the extracellular domain of TNFR‐2 linked to the Fc portion of a human IgG1 over a 6‐week period. The result suggests both interleukin‐1β and tumor necrosis factor‐α as potential target treatments for periodontal disease. Therefore, targeting other inflammasome components prior to interleukin‐1β secretion may provide alternative paths for modulating the periodontal host response.

#### 6.3.2 Interleukin‐18

The relevance of interleukin‐18 in disease pathogenesis is less explored compared with interleukin‐1β, translating into fewer trials evaluating interleukin‐18 blockade as a strategy for therapy. A small sample study in diabetes concluded that although the usage of GSK1070806, an anti‐interleukin‐18 monoclonal antibody, was well tolerated, inhibition of interleukin‐18 did not lead to any improvements in glucose control.^171^ Evaluation of therapeutics targeting interleukin‐18 for treating periodontal disease still requires further biological justification.

Additional inflammasome inhibitors targeting reactive oxygen species, caspase‐1, NLRP3 and pyrin are under clinical trials for treating several diseases (Tables 2, 3, 4). The data we evaluated support a role for inflammasomes in periodontal disease. Therefore, testing of their therapeutic efficacy in periodontal disease is merited.

## 7 SUMMARY AND CONCLUSIONS

The literature supports that inflammasomes have a central role for microbe and damage sensing within the innate immune system and, as such, manipulation of its components is shown to be therapeutically beneficial. Aberrant inflammasome activation is observed in diseased tissues derived from clinical and preclinical studies. In autoinflammatory diseases such as Crohn's disease and Behcet disease, genetic variations alter the protein function by auto‐activating or lowering the threshold for activation of inflammasomes that appears to affect clearance of pathogens and the integrity of the epithelial/mucosal barrier that normally protects the host. Interestingly, the highlighted loci associated with a periodontal phenotype in a recent genome‐wide association study analysis included genes associated with immune response and epithelial barrier function.^107^ In inflammatory diseases, such as atherosclerosis, diabetes and obesity, there is a chronic stimulus that primes and induces inflammasome activation. The involvement of inflammasome components in periodontal health and subtypes of disease are in the early stages of exploration. Are components shown to be expressed highly in diseased tissues the ones that will affect disease development in therapeutic approaches? Is there an inflammasome pathway that can hamper periodontal disease development in patients? Are there subtypes of periodontal disease that require different inflammasome targeting? The data currently indicate that several inflammasome components are expressed at higher levels in saliva, gingival crevicular fluid and periodontal tissues. Individuals with autoinflammatory disease may have different biological pathways that lead to periodontal disease. It is unknown if the biological pathways that lead to aberrant inflammasome activation in chronic diseases also affect the periodontal host response. There is strong impetus for further clarification of the role of inflammasome pathways as targets for treating periodontal disease.
